# Modulating Protein–Protein Interactions by Cyclic and Macrocyclic Peptides. Prominent Strategies and Examples

**DOI:** 10.3390/molecules26020445

**Published:** 2021-01-16

**Authors:** Rosario González-Muñiz, María Ángeles Bonache, María Jesús Pérez de Vega

**Affiliations:** Instituto de Química Médica (IQM-CSIC), Juan de la Cierva 3, 28006 Madrid, Spain; angelesbonache@iqm.csic.es (M.Á.B.); pdevega@iqm.csic.es (M.J.P.d.V.)

**Keywords:** cyclic peptides, macrocyclic peptides, protein–protein interactions, computational design, biochemical methodologies, synthetic strategies, cell penetrating conjugates, peptoids

## Abstract

Cyclic and macrocyclic peptides constitute advanced molecules for modulating protein–protein interactions (PPIs). Although still peptide derivatives, they are metabolically more stable than linear counterparts, and should have a lower degree of flexibility, with more defined secondary structure conformations that can be adapted to imitate protein interfaces. In this review, we analyze recent progress on the main methods to access cyclic/macrocyclic peptide derivatives, with emphasis in a few selected examples designed to interfere within PPIs. These types of peptides can be from natural origin, or prepared by biochemical or synthetic methodologies, and their design could be aided by computational approaches. Some advances to facilitate the permeability of these quite big molecules by conjugation with cell penetrating peptides, and the incorporation of β-amino acid and peptoid structures to improve metabolic stability, are also commented. It is predicted that this field of research could have an important future mission, running in parallel to the discovery of new, relevant PPIs involved in pathological processes.

## 1. Introduction

Medicinal chemistry programs focused on the search for protein–protein interaction (PPI) modulators are pursued by either academic and pharma/biotech companies [1,2]. The motor of this interest is the wide implication of PPI networks, the so called interactome [3], as regulators and executors in almost all biological processes, including cell signaling, survival, and proliferation, as well as pathogen–host recognition, among others. Only a few therapeutically relevant PPIs have been identified to date, so the interactome remains mainly unexplored and could be the foundation to design new drugs for unmet medical needs [4].

The interaction between two proteins normally involves a large and relatively flat surface, although certain nodes (hot-spots) could contribute in a special manner to maintain the protein–protein contact [5,6]. In this sense, peptides constitute a primary source of modulators for disrupting/stabilizing PPIs since they can be prepared in different length and amino acid composition by relatively simple, routine methods [7]. Peptides normally are endowed with high flexibility, resulting in low selectivity, and poor metabolic stability. Along with the backbone modifications, cyclization is one of the most popular structural changes to improve pharmacokinetics in peptides [8,9]. In this context, cyclic and macrocyclic derivatives attract much attention as valuable scaffolds for targeting PPIs [9,10]. They represent a halfway between biologic and small-molecule drugs, more accessible synthetically than the former, facilitating medicinal chemistry programs, and bigger in size than the latter (3–5 times larger), allowing them to interact with wide surface areas, which usually are needed for strong PPI interactions [11,12]. In fact, structural studies on cycle/macrocycle-protein complexes revealed that their surfaces are similar in size to native PPI interfaces and antibodies [13]. In addition, cyclic and macrocyclic peptides have conformational rigidity that control defined 3-D orientations of amino acid side-chains, and have more resistance to proteases than linear peptide counterparts. For these reasons, they are considered promising candidates for the development of chemical probes and novel therapeutics [14,15]. Among natural product macrocycles and cyclic peptides, there are about 68 marketed drugs and 40 more under clinical development [16,17], including the well-known examples cyclosporine A, caspofungin and daptomycin.

Nonetheless, there are some drawbacks for the implementation of cyclic and macrocyclic peptides as a more general strategy to PPI modulation and their translation into suitable drugs. Among them, the challenging synthesis, with a few, fast general methods to obtain large cycles in a convergent manner [18,19,20], the difficulties to get crystal structures of their complexes with interacting proteins, and their impermeability to the cell membrane preventing entry to intracellular targets [21].

More than an exhaustive review on cyclic and macrocyclic peptides described to interfere within PPIs in the last 10 years, this review wants to call the attention on some recent, innovative strategies and outstanding examples around this topic. The design directed by computational methodologies or based on PPI X-ray structures, the isolation from natural sources, and both biochemical and synthetic approaches to cyclic/macrocyclic peptides are considered here. The conjugation with CPPs to facilitate cell permeation, and the properties of some peptoid analogues are also mentioned. Stapled-type cyclic peptides for stabilizing helical conformations will marginally covered in this compendium because the high number of revisions appeared in the contemplated time lapse [22,23,24,25,26,27].

## 2. Main Methodologies to Cyclic and Macrocyclic Peptides

Amide head to tail, head/tail to side-chain, and side-chain to side-chain cyclization, along with disulfide formation, are conventional approaches normally used to generate mono- and polycyclic peptides [28]. Additionally, the incorporation of non-proteinogenic amino acids containing different functional groups is being increasingly explored to facilitate other ways of cyclization (i.e., stapled peptides) [23]. The development of various synthetic methodologies to generate macrocycles through the cyclization of peptides with different organic frameworks is also the subject of much attention in recent times [10]. In this section, we provide some wide ranging examples of bioactive cyclic and macrocyclic peptides and the different strategies to reach them.

### 2.1. Computer-Assisted and X-ray-Based Design

**Figure 1:**
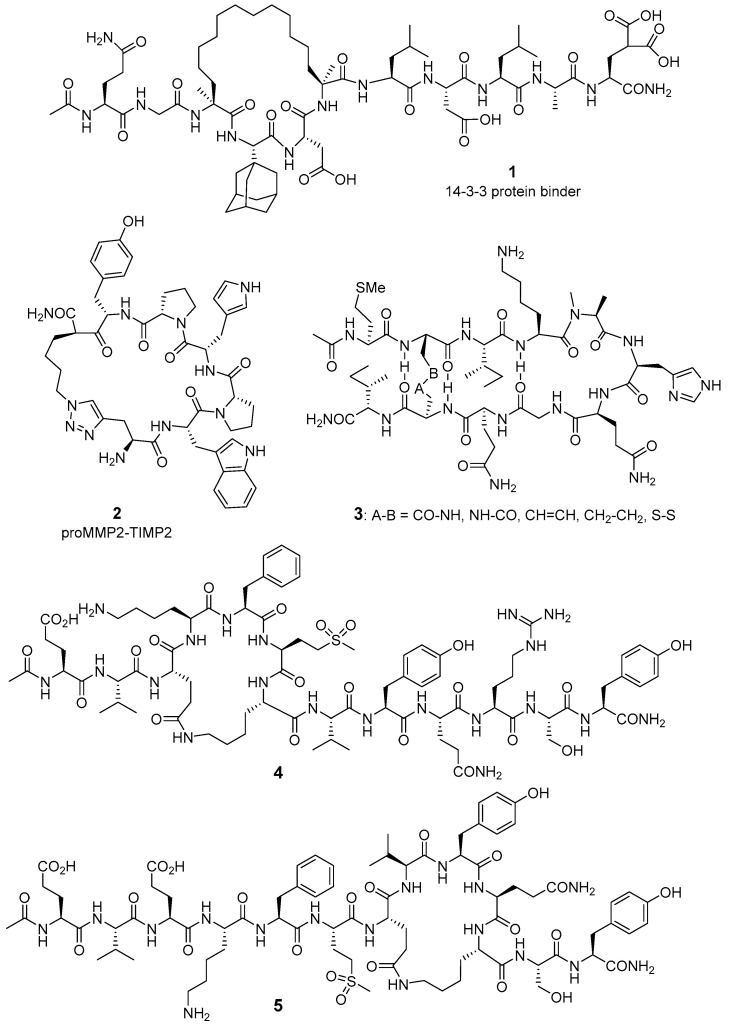
Cyclic peptides obtained after computer simulations or designed from protein–protein interaction (PPI) X-ray structures.

Computational techniques, combined with solid-phase peptide synthesis and different biophysical characterization methods, have been used to predict non-discovered PPIs, to identify PPI surfaces, to validate experimental results, and to virtually screen libraries of linear and cyclic peptides. Thus, computer simulation through structure-based design tools is in the origin of cyclic peptides that behave as PPI modulators, with several examples of computational screening approaches recently reviewed [29,30,31]. To exemplify this topic, from a model macrocyclic peptide able to bind 14−3−3 proteins, Kruger et al. generated a virtual library of >1400 stapled cyclic peptides including non-natural amino acids [32]. The interaction of 14−3−3 proteins with the transmembrane receptor aminopeptidase N (APN) prompts an intracellular signaling cascade, activating the expression of matrix metalloproteinases (MMPs) [33], which are important modulators of the extracellular matrix, and are upregulated in rheumatoid arthritis and metastatic cancers. Therefore, the inhibition of extracellular PPIs involving 14−3−3 proteins can decrease MMP transcription levels. Library filtration through docking scores and combination of the best modifications allowed the identification of higher affinity peptide derivatives, like compound **1** (Figure 1). This hydrocarbon-stapled peptide displays higher affinity than the model macrocyclic peptide for 14−3−3ζ protein, and is able to significantly reduce MMP1 transcription. X-ray crystallography confirmed then the binding mode, thus verifying previous docking results [32].

As an alternative approach to target metalloproteinases actions, the group of Ming Xue designed an epitope-targeted library of cyclic peptides to interfere within the interaction between tissue inhibitor of metalloproteinases-2 (TIMP2) and proMMP2 [34]. Cyclic peptide derivative **2**, with a triazole stapled linker, induces a concentration-dependent decrease in MMP2 activity in culture media, acting directly and selectively on MMP2 (not affecting MMP9). The importance of the cyclized structure is demonstrated by the inactivity of the corresponding linear sequence in the same experiment. Compound **2** also decreases cell mobility in a wound-healing scratch assay, and this activity correlates with the measured K_D_ of its interaction with proMMP2, supporting a mechanism of action involving the indicted PPI.

The vascular endothelial growth factor (VEGF) is one of the most important factors promoting angiogenesis, the process of formation and proliferation of blood vessels. Angiogenesis plays an important role both in physiologic and pathologic situations, like for instance cancer and rheumatoid arthritis [35]. VEGF exerts its proangiogenic activity through the interaction with its transmembrane receptors, which has tyrosine kinase activity. Despite the interest in small-molecule tyrosine kinase inhibitors [36], targeting the extracellular domain of VEGF receptor 1 (VEGFR1) to hamper its interaction with VEGF could constitute an alternative strategy to modulate this proangiogenic system. Based on the X-ray structure of VEGF–VEGFR1 complex, and taking as model the amino acid sequences of VEGF identified as key hot-spots for its interaction with VEGFR1, Pérez de Vega and co-workers designed conformationally constrained peptide analogues for hampering the VEGF–VEGFR1 interaction; therefore, for blocking the cascade of biological events triggered by this protein–protein interaction [37,38,39]. Different mutagenesis and structural studies permitted the identification of two main hot-spots, at the interaction interface of VEGF with VEGF1, namely fragment VEGF_81–91_, which is part of a β-hairpin structure, and fragment VEGF_17–25_, located at the N-terminal and having α-helical conformation. With the aim of mimicking the 3D structure adopted for the VEGF hot-spots native sequences, cyclic constrained 10 to 13-mer peptide derivatives were designed (Figure 1, examples **3**–**5**). The strategy involved the crosslinking of amino acid side-chains keeping unaltered the residues reported as important for the molecular recognition. To mimic the native β-hairpin of VEGF_81–91_ fragment, different type of bridges were generated to link together the two strands, in particular hydrocarbon-, disulfide-, and amide-linkers (CO-NH and NH-CO) [37,38]. The restriction with the NHCO bridge afforded the best cyclic peptide in the series (IC_50_ = 87.6 μM). Concerning the second hot-spot, VEGF_17–25_, and to fix the α-helix, amide bridges were formed from the amino acid side-chains of conveniently situated Glu and Lys residues, at relative positions *i* and *i* + 4. Analogues of both VEGF_17–25_ and Vammin_69–80_, the homologue fragment of an antiangiogenic VEGF-related protein from snake venom, were prepared [39]. Vammin-derived cyclic peptide **4**, having the conformational restriction closer to the N-terminus of the sequence, compared to regioisomeric analogue **5**, exhibits the best IC_50_ value (36 μM).

### 2.2. Cyclic Peptides of Natural Origin

**Figure 2:**
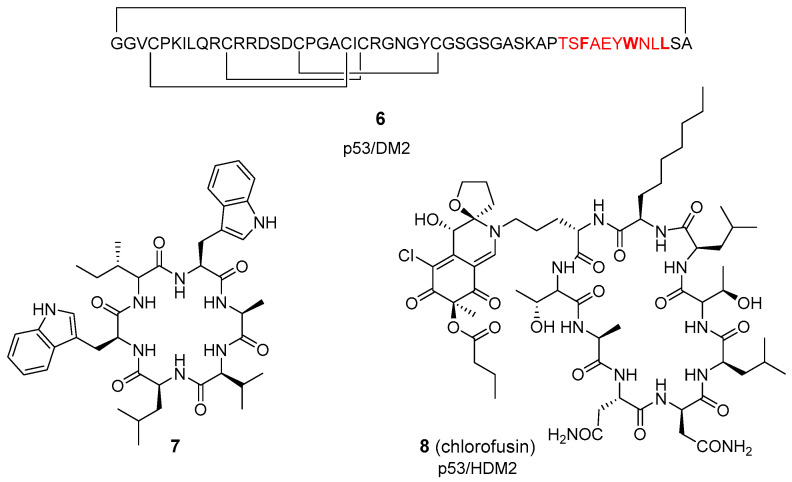
Selected examples of cyclic peptides isolated from natural sources or obtained after modification of naturally-occurring cyclopeptides.

Isolation from natural sources is a complementary technology to feed screening programs for PPI modulation. Plant-derived cyclotides are disulfide-rich cyclic peptides with extraordinary resistance to thermal, chemical, and enzymatic degradation, and good cell-penetrating ability, and therefore, good candidates to modulate intracellular PPIs [40,41]. Engineering the metabolically unstable N-terminal fragment of p53 (Figure 2, labeled in red) into a cyclotide scaffold results in cyclic derivative **6** (MCoTI-I) as an efficient antagonist of the intracellular p53 degradation [42]. This compound is highly stable in human serum, binds to HDM2 protein with low nanomolar affinity, and shows both in vitro and in vivo cytotoxic activity in wild-type p53 cancer cell lines. In LNCaP cells, cyclic peptide **6** activates the p53 tumor suppressor pathway by disrupting the p53−Hdm2 complex and inducing G1/S cell cycle arrest. A number of naturally-occurring cyclopolypeptides of marine origin, like proline-rich derivatives [43], and small Trp-derived peptides (**7**, Figure 2) [44], also display a broad range of pharmacological activities, including antitumor, antibacterial and antiviral actions. Despite that these marine cyclic peptides could be useful scaffolds for modulating challenging PPIs, to the best of our knowledge there is no information about their specific PPI targets.

M. Moore’s group described the isolation from *Microdochium acaespitosum,* and ulterior synthesis of the natural cyclopeptide chlorofusin (**8**, Figure 2) [45]. These cyclic nonapeptides, which bear a chromophore on the Orn side-chain, blocks the p53–MDM2 PPI (IC_50_ = 4.6 µM) [46], through direct contact with HDM2 protein (K_D_ = 4.7 µM), as demonstrated by surface plasmon resonance (SPR) spectroscopy. More recently, a simpler 4-methylphenyl triazolyl derivative at the Orn side-chain was also described to prevent this PPI, but with one order of magnitude lower activity than the natural product [47]. Fortunately, the synthetic intermediates leading to this latter chlorofusin peptide derivative allowed the identification of new non-peptide inhibitors of the p53–MDM2 interaction.

### 2.3. Biochemical Approaches to Cyclic Peptides

**Figure 3:**
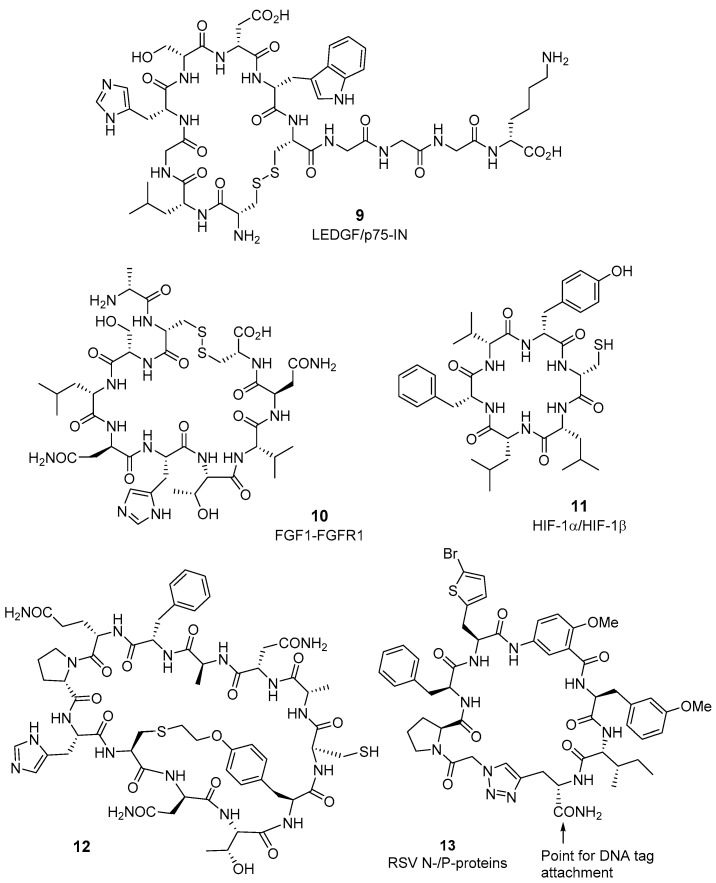
Cyclic/macrocyclic peptides discovered after application of biochemical technologies.

Different biochemical technologies have been developed to assist in the generation of cyclic/macrocyclic peptide combinatorial libraries and their evaluation in high-throughput screening (HTS) systems as modulators of PPIs (recently reviewed in [10,48]). These techniques and some selected examples are discussed below, and depicted in Figure 3.

The phage display technique, a process in which large, highly diverse libraries of peptide/protein variants are generated on the phage surface, is a dominant tool in mapping protein–protein contacts and the search for PPI modulators [49,50]. Thus, disulfide bridged cyclic peptides were identified by phage display as the first inhibitors of HIV integrin (IN) and the cellular cofactor lens epithelium-derived growth factor (LEDGF/p75). Synthetic sequences corresponding to the identified peptides, like **9** (Figure 3), block HIV replication in different cell lines, while scramble analogues do not. The results indicate that the Trp residue is crucial for the interaction with LEDGF/p75 protein and suggest that the LEDGF/p75–IN interaction could be important for HIV replication and an emerging therapeutic target [51]. Fibroblast growth factor receptor 1 (FGFR1) is overexpressed in lung and breast cancers and therefore drugs targeting FGF1/FGFR1 PPI could be an alternative to small-molecule tyrosine kinase inhibitors for handling FGFR1-dependent cancers. In this respect, the group of Jacek Otlewski described the selection of cyclic peptides able to disrupt the FGF1/FGFR1 interaction prepared by phage display [52]. The most potent cyclic peptide **10**, but not its linear peptide equivalent, is able to inhibit the FGFR1 downstream signaling and the FGF1-induced cell proliferation in cell expressing FGFR1. This peptide could be the starting point to new molecules targeting the FGF1/FGFR1 interaction for fating against FGFR1-overexpressing cancers. 

Macrocyclic organopeptide hybrids (MOrPHs), a strategy developed by Rudi Fassan’s group, conjugate non-proteinogenic synthetic moieties into genetically encoded peptide frameworks [53]. The method consisted in the incorporation of an N-terminal *O*-alkyne-containing Tyr residue into intein-fused polypeptides, followed by chemoselective triazol formation with and azide/hydrazide-containing organic moiety. The thioester transient bond at the connection with the intein makes possible the cyclization of the recombinant peptide to organopeptide macrocycle through a hydrazide bond. Another very recent example describes the generation of 10^5^- to 10^8^-member libraries constrained through non-reducible thioether bridge [54]. Further screening against different protein–protein interaction, led to the identification of potent peptide macrocycles to disrupt the Keap1/Nrf2 (best component K_D_: 40 nM), a PPI associated to the upregulation of cytoprotective enzymes of interest for inflammatory processes, and the interaction between Hedgehog interacting protein (HHIP) and Patched-1 (PTCH1) transmembrane receptor (K_D_: 550 nM), of interest for treating various human malignancies [54]. 

The ribosomal synthesis of cyclic peptides in bacteria is another valuable approach toward big peptide libraries. As representative examples, the head-to-tail cyclohexapeptide **11** (Figure 3), identified from a big library generated using split-intein mediated protein splicing (SICLOPPS) [55], behaves as an inhibitor of the HIF-1α/HIF-1β PPI in vitro and prevents HIF-1-mediated hypoxia in cells, altering transcriptional responses to hypoxia [56]. Similarly, cyclo-SGWTVVRMY was discovered through the SICLOPPS technology as a disruptor of the dimerization of the C-terminal binding protein transcriptional repressor (CtBP1/CtBP2), involved in epigenetic regulation and in the proliferation and migration of cancer cells [57]. Ribosomal derived peptide sequences can be also macrocyclized through chemoselective reaction between an unnatural amino acid, the *O*-(2-bromoethyl)-tyrosine, and a cysteine residue, which combined with the SICLOPPS strategy allow the preparation of macrocyclic peptides (i.e., compound **12**, Figure 3), which could also be of future interest toward PPI inhibitors [58]. 

Yeast surface display, alone or in combination with phage display, starts to be an approach for the quick identification of protein binders, with only a few examples to express cyclic peptide derivatives nowadays. Thus, genetically encoded libraries generated by using yeast surface display serve to identify cyclic peptides that bind to lysozyme, interleukin-17 (IL-17) and IL-23 [59,60], and help to envisage this technique as a future strategy toward peptide drug leads and inhibitors of relevant PPIs. Another attractive, completely in vitro method for library production is the mRNA display, which allows the insertion of non-natural amino acid residues and peptide cyclization [61,62], though the application to find PPI binders need to be explored yet. 

As for small-molecule libraries [63], the incorporation of a DNA sequence during the synthesis of combinatorial peptide libraries constitutes another valuable approach for the production of big library pools, isolation of peptide sequences with high affinity for a given protein, and simple determination of peptide primary structure by sequencing the encoding DNA [64]. In this sense, the group of Z. Zhu described the synthesis and biological evaluation of a DNA-encoded macrocyclic peptide library with 2.4 × 10^12^ components, by combining proteinogenic and non-proteinogenic amino acid residues [65]. This library was tested as inhibitors of the PPI between N- and P-proteins of the respiratory syncytial virus (RSV), important for replication and considered an innovative target to fight against the infection caused by this virus. After identification of the most interesting sequence binders, selected cyclic peptides were resynthesized without the DNA tag, using solid-phase methodologies, and reevaluated. Compound **13** (Figure 3) is one of the best confirmed hits, with good ability to bind to N-protein (affinity selection−mass spectrometry assay) and to disrupt the interaction of RSV N- and P-proteins (pIC50 6.98, time-resolved fluorescent resonance energy transfer assay) [65]. The activity is much higher for this cyclic peptide than for the corresponding linear analogue, suggesting that the reduced mobility of **13** could result in lower entropic cost for binding. 

### 2.4. Synthetic Strategies to Cyclic Peptide Libraries

Regarding chemical approaches, in recent years, there is a continuous development of strategies for the preparation of cyclic peptides, their diversification, and their presentation on tagged-resins, all with the main objective of allowing a rapid identification of active peptide derivatives. In addition, there is an important progress in different stratagems for obtaining macrocyclic products, which comprise a peptide sequence and a more or less extensive, non-peptide organic region that could serve either to modulate the activity of the peptide or to facilitate the macrocyclization process [66].

**Figure 4:**
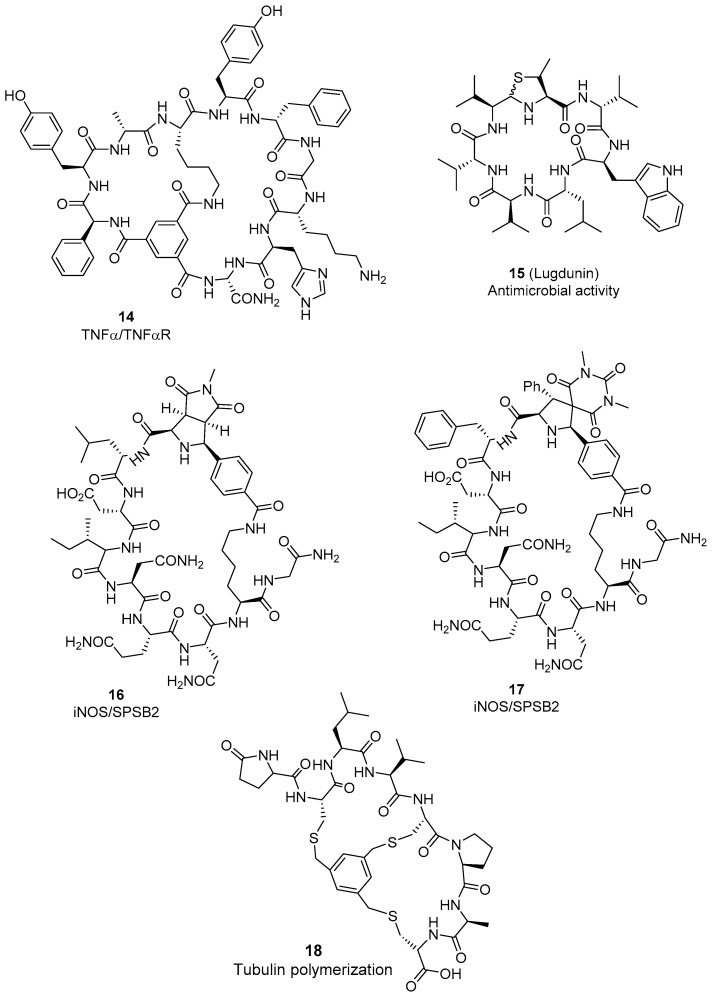
Selected components of totally synthetic macrocyclic peptide libraries with PPI disrupting properties.

The group of Dehua Pei designed and synthesized a large combinatorial library of bicyclic peptides (6.6 × 10^13^ components) and screened it against TNFα/TNFαR PPI [67]. The format of the library is one bead−two compound (OBTC), each bead containing a unique bicyclic peptide and the corresponding linear analogue, as encoding tag for facilitating hit identification. This library, combining proteinogenic and unnatural amino acids, contains a planar organic scaffold, trimesic acid, to foster an overall planar shape of the bicyclic peptide for facilitating the interaction with flat protein surfaces. One component of this library, named anticachexin C1 (**14**, Figure 4), is a potent non-protein TNFα inhibitor (K_D_ = 0.45 μM) [67]. 

New methods toward cyclic peptides formed via linkage between amino acid side-chain functional groups and reactive organic moieties have recently been reported. Thus, Luo et al. described the synthesis of 1,4-di-nitroimidazole-derived peptides formed by electrophilic bioconjugation of Cys and/or Lys side-chains and 4-nitroimidazol derivatives [68]. The method is chemoselective, controllable under different reaction conditions, allowing easy access to macrocyclic peptide with complex ring structures, and selective protein modification. Another relevant cyclization method was described by the group of Xuechen Li [69], which prepares isoindole-bridged cyclic peptides through simultaneous reaction of the amino group of Lys side-chain (or the N-terminal NH_2_) and the thiol group of Cys side-chains with orthophthalaldehyde (OPA). This transformation takes place in an efficient manner in aqueous buffers and can also be combined with other cyclization methods, like native chemical ligation. In addition, OPA-derived cyclic peptides can react with *N*-maleimide derivatives, containing biomolecules or fluorophore probes for further bioconjugation.

The use of multicomponent reactions (MCRs) to facilitate the macrocyclization of peptides is also being widely applied, as described in a recent comprehensive review [70]. In this respect, Dömling et al. described a library of indole-based macrocyclic p53–MDM2 antagonists, using an Ugi macrocyclization from 12 α-ω-amino acids and indole-3-carboxaldehydes [71]. On the other hand, the generation of imine intermediates for further diversification also merits special attention. Thus, the group of Phil Baran described the imino macrocyclization in aqueous media of non-ribosomal peptides [72]. Stating from linear peptide aldehydes with certain propensity to autocyclize to the corresponding imine derivatives, they drive imine trapping through intermolecular Strecker protocols and reductive amination procedures, or intramolecular entrapping using appropriate N-terminal amino acids (tetrahydro-β-carbolines from Trptetrahydro-1H-imidazo [4,5-c]pyridines from His, and thiazolidine and oxazolidine from Cys and Thr). The described procedures accept a wide variety of linear peptides (5 to 10 amino acid residues), which can include functionalized side-chain residues (Arg, Asp, Gln, Cys(StBu), Lys, Tyr). The cyclization process can be modulated through the incorporation of N-Me groups and non-natural D-residues. This versatile procedure was applied to the preparation of different naturally-occurring macrocyclic peptides and analogues, like lugdunin (**15**, Figure 4), an antimicrobial peptide with activity against Gram-positive bacteria (i.e., Staphylococcus aureus) [72]. Another approach that used cyclic imine peptides as key intermediates for further molecular expansion is the PepNats strategy, recently described by the group of Herbert Waldmann [73]. In this case, for exploratory studies, all initial linear peptides incorporating a Gly residue at *N*-terminal, and a Lys(Mtt) residue at n-1 position, which upon orthogonal Mtt removal and coupling of *N*^ε^-Lys with 4-formylbenzoic acid affords the aldehyde group for the Schiff base formation. Trapping the imine moiety with different dipolarophiles (1,3-dipolar cycloaddition), through previous azometine ylide generation, afforded different natural product-inspired cycloadducts. The entire process can be conducted on solid-phase and is versatile regarding peptide sequence, enabling the stereoselective preparation of libraries of diverse heterocycle-containing peptide macrocycles. NMR studies demonstrated that the conformational preferences in solution of these PepNats are dependent on the configuration and structure of the heterocycle, factors that also impact their activities as PPI modulators. Inducible nitric oxide synthase (iNOS) is a key mediator of immune activation and inflammation, with overexpression or dysregulation in pathologies like osteoarthritis, sepsis, cancer, neurodegeneration, and various types of pain. The lifespan of iNOS is regulated by the proteasome after ubiquitination by the E3 ubiquitin ligase complex, being SPSB2 one of the adaptor proteins. In cancer, production of NO could contribute to kill tumor cells, therefore, disruptors of the SPSB2-iNOS interaction could led to alternative antitumor agents. Application of the PepNat strategy to the modulation of the iNOS-SPSB2 PPI led to the discovery of di-pyrrolidine and pyrrolidinyl-spirobarbiturate macrocyclic peptides **16** and **17** (Figure 4), with low nanomolar potencies in the binding tor SPSB2 (IC_50_ = 10 and 3.8 nM, respectively). Therefore, the imine-based synthetic methodologies open the opportunity to apply imine-derived macrocyclic peptides in the search for modulators of other therapeutically relevant PPIs.

Other efforts have been directed to complex bicyclic peptide structures. Thus, the group of Craig A. Hutton reported a small library of bicyclic peptide derivatives as analogues of celogentin C, a natural product that inhibits tubulin polymerization. They ideated the formation of the bicyclic core by using a *tris*-(bromomethyl)benzene moiety and three Cys residues conveniently positioned within the linear peptide precursor. SAR studies show that Leu3, Val4, and Pro6 are essential residues for activity. In the most active compound, **18** (IC_50_ = 2.2 μM), the Arg7 residue in celogentin C is replaced by Ala (Figure 4). Macrocyclic peptide **18** shows enhanced activity over the antitumor drug vinblastine [74]. Using a similar strategy, Timmermann’s group discovered, via HTS of partially randomized libraries, new bicyclic RGD peptides with nanomolar affinity, and selectivity for integrin αvβ3, as antitumor and diagnosis agents [75]. The formation of the bicyclic peptide derivatives was carried out by reaction l- or d-Cys(c) residue with 1,3,5-*tris*(bromomethyl)benzene, combining the universal integrin-binding sequence Arg-Gly-Asp (RGD) in one loop, and a randomized sequence XXX (X = one of 18 canonical l-amino acids) in the second loop.

### 2.5. Cell Penetrating Cyclic Peptides

To increase the permeability of these normally big molecules, the conjugation with cell-penetrating peptide (CPP) and the facilitation of active transport have been studied [20,47]. Two main ways have been described for the integration of cyclic peptide cargos with cyclic CPPs, the conjugation through different types of linker and the generation of fused bicyclic peptides [76].

**Figure 5:**
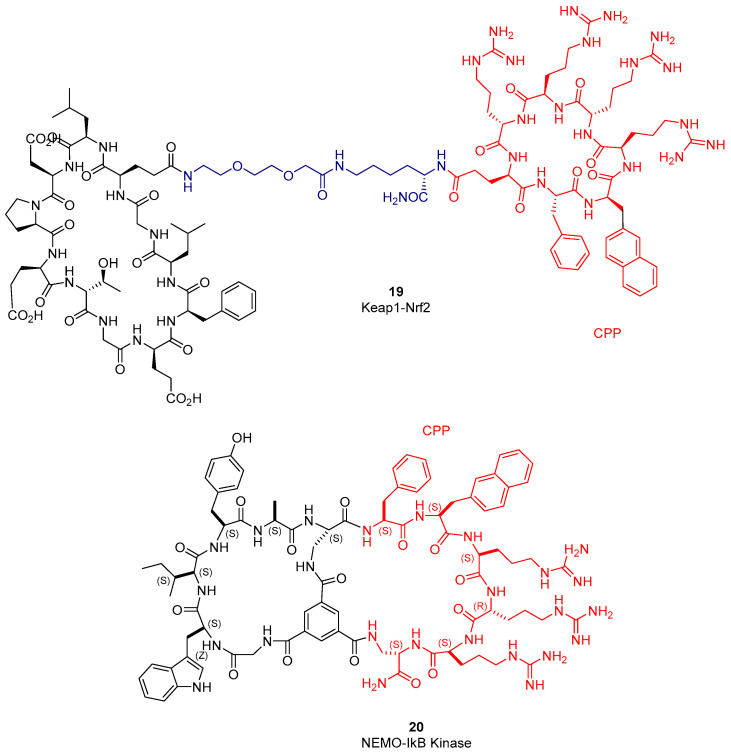
Selected examples of cell-penetrating peptide (CPP)–cyclic peptide conjugates and bicyclic-merged peptides.

Within the first strategy, the group of Dehua Pei described the conjugation of a cyclic peptide inhibitor of Kelch-like ECH-associated protein 1 (Keap1)–nuclear factor erythroid-2 (Nrf2) PPI and a cyclic poly-Arg CPP, covalently linked through flexible connectors [77]. Bicyclic peptide **19** (Figure 5) retains Keap1-binding affinity (IC_50_ = 48 nM), is quite stable against proteolytic degradation, penetrates mammalian cell membranes, and activates gene transcription mediated by Nrf2. A similar, recent work affords important SAR on the structural requirements of the KEAP1-NRF2 PPI, and the conjugation of NRF2-derived cyclic peptides to different CPPs increases the in vitro activity compared to their unconjugated analogues [78].

As a proof-of-concept to enhance the limited cell permeability of stapled peptides, some impermeable peptidyl inhibitors of MDM2/p53 and β-catenin/TCF PPIs were conjugated with cyclic CPPs [79]. This approach ensured peptide conjugates with antiproliferative activity, decreasing the viability of SJSA-1 and SW480 tumor cell cultures.

In the second approach, Pei’s group merges randomized cyclic hexapeptides, composed of natural and non-proteinogenic residues, with diverse CPP sequences to generate a large library of bicyclic peptides [80]. This library was checked for inhibition of the NF-κB essential modulator (NEMO)-IκB kinase interaction. They identify cell-permeable bicyclic peptide **20** (Figure 5) as the most potent binder of NEMO protein reported to date (IC_50_ = 1 µM). This bicyclic peptide inhibits de NF-kB signaling and shows anticancer activity in A2780 and CP70 tumor cell lines [80]. 

Another prominent strategy for inhibitors of intracellular PPIs is grafted peptides, combining in a unique cyclic molecule a protein epitope and an Arg-rich peptide. As an example, the helical p53 epitope embedded together with helical peptides containing up to six Arg residues, led to inhibitors of the p53–HDM2 interaction [81]. The best peptide, shows high binding affinity for HDM2, cell membrane penetration, and antiproliferative activity in vitro (HCT116 and LnCap cells), suggesting a p53-dependent in vitro activity.

All these approaches could be applicable to the discovery of cell-permeable peptides against other intracellular PPI targets. In addition, the identification of new CPPs is pursued to discover shuttling vectors for peptide cargo transportation. In this respect, SARS-CoV2 virus, the etiological causative of COVID-19, needs to infect animals or humans to replicate, as other viruses, and as a consequence it should have CCPs in its proteome. A recent computational study analyzed the SARS-CoV-2 proteins and identified >300 sequences with potential CPP ability and involved in important processes like protein–protein interactions and homo/hetero-oligomer stabilization [82]. Further filtering allowed the identification of peptide sequences with no antigenic or allergenic properties and resistant to main groups of proteases. Some Cys-rich CPPs from helicase (NSP13), which could fold into cyclic peptides in endosomes, have also been envisaged. All these sequences could constitute the starting point for the discovery of innovative CPPs, which could have biological activity by themselves, or serve as transporters to permeate other bioactive peptides into the cell.

### 2.6. Photoswitchable Cyclic Peptides 

Photoswitchable peptides PPI inhibitors are molecules that only adopt their active/inhibitory conformation when exposed to light at certain wavelengths. Olalla Vazquez and Lea Albert recently summarized the state-of-the-art on photoswitchable peptides for the control of biological functions [83]. 

**Figure 6:**
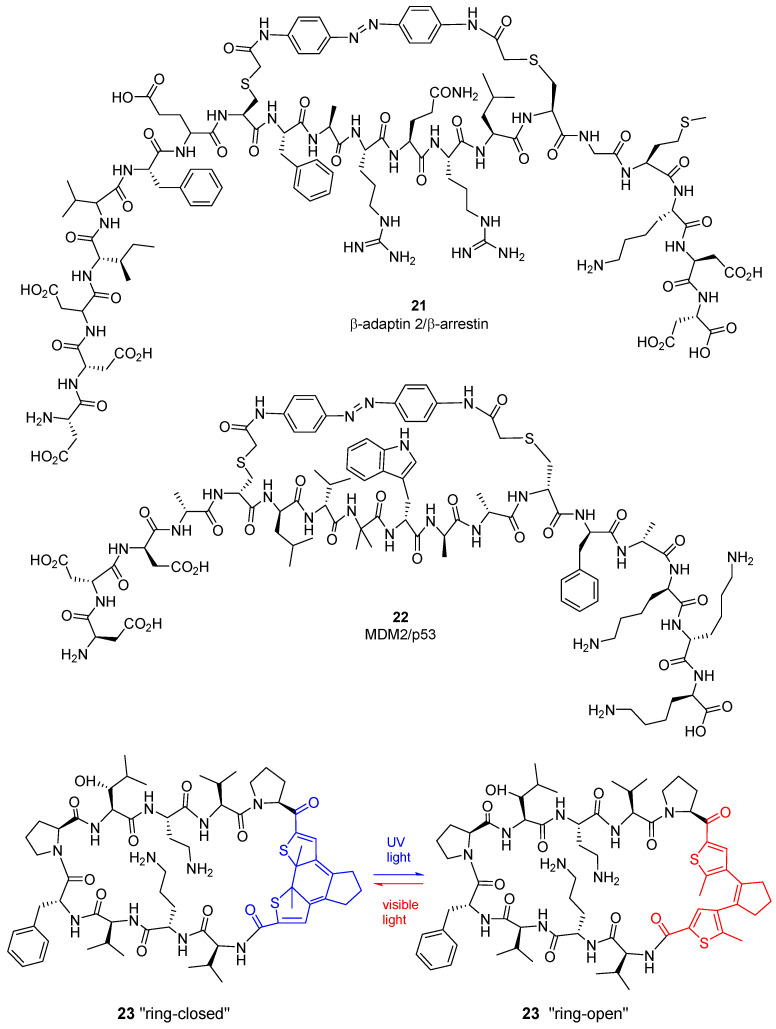
Selected examples of photoswitchable cyclic peptides.

The group of Ernest Giralt described a series of 20-mer azobenzene-crosslinked peptides to fine-tune their interaction with β-adaptin 2, a key protein involved in clathrin-mediated endocytosis through its interaction with β-arrestin [84]. This study concludes that the helix stabilization by the photoswitchable moiety or by incorporation of Aib residues is not a requirement for good inhibitors. The best peptide in this series, **21** (Figure 6), has i, i + 7 crosslinking and shows a Ki^off^/Ki^on^ >10 (micromolar range). The same group defined new photoswitchable peptides for interfering in Bcl-xL–Bak and MDM2–p53 PPIs [85], involved in apoptosis. In the last case, they prepare photoswitchable retro-enantio p53-derived peptides, with partial α-helical structure. Peptide **22** (Figure 6) interact with MDM2, showing low activity at the dark and a 10-fold strong binding after UV light exposure (380 nm illumination).

A different approach has been described by the Urlich’s group [86], using a photoswitchable diarylethene moiety to stabilize the β-hairpin secondary structure of the natural membranolytic peptide gramicidin S (**23**, Figure 6). The library of peptides was tested for their antibacterial and antitumor activity. In general, these peptides show low hemolytic cytotoxicity, especially for hydroxyleucine-containing derivatives. Compound **23** shows selective antimicrobial activity against Gram+ bacteria and cytotoxicity in different cancer cell lines, and was active in doxorubicin-resistant tumor cells [86]. A small library of related photoswitchable analogues of gramicidin S were studied for their embryotoxicity in the two photoforms, revealing higher toxicity for ring-open isomers [87].

### 2.7. β-Amino Acid- and Peptoid-Containing Cyclic Peptides

Other strategy for constructing peptide-based PPI inhibitors is the modification of the backbone structure, by extension (incorporation β-amino acid) or by shift of side-chains to nitrogen atoms (peptoids). Oligomers that contain only β-amino acids (β-peptides) or both natural α-amino acids and unnatural β-amino acids (α/β-peptides) normally display high resistance to proteolytic degradation [88], can mimic natural α-helices, and are capable of modulate helix-mediated PPIs [89]. The synthesis of such peptides can be considered practicable, based on standard SPPS protocols, and is facilitated by a number of commercially available β-amino acids, which also could be obtained through quite simple synthetic methods [90]. Due to their high conformational stability, β-peptides are shorter than α-peptides but can achieve a similar degree of folding. The use of this type of backbone-modified peptides as inhibitors of PPIs has previously been revised [91], with important examples targeting Bcl-2–2BH3 domain [89,92], VEGF–VEGFR1 [93], and p53–MDM2 [94], among others. As a recent example of α/β-hybrid peptides, the group of Douglas Fairlie reported a few peptides, based on a stapled Bim BH3 structure, with enhanced α-helix stability (CD), high resistance to proteolytic degradation (>100), and comparable ability to neutralize anti-apoptotic protein function, in a cellular milieu, to the parent stapled α-peptide [95]. 

**Figure 7:**
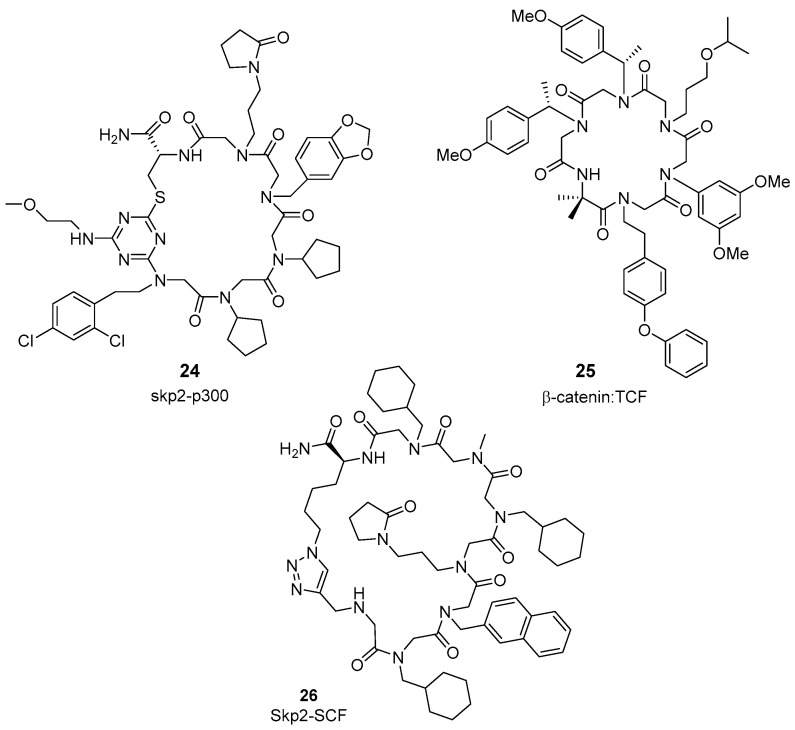
Peptoid-based inhibitors of PPIs.

Other particular promising class of foldamers is the peptoid structure, composed of N-substituted glycine peptidomimetics. In peptoids side-chains are moved from the α–carbon to backbone amide nitrogen. This backbone modification provides high protease resistance, although the positioning of key interacting residues in the right place is sometimes a challenge. Peptoid-based inhibitors, can be exemplified by PPI inhibitors of VEGF–VEGFR [96], apoptotic protease-activating factor 1 (Apaf-1) [97], and skp2–p300 [98]. Thus, macrocyclic peptoid **24** (Figure 7) binds to Skp2 protein and interferes with the Skp2/p300 interaction, a PPI with potential to develop new anticancer drugs. Skp2 mediates the degradation of cell cycle proteins and suppresses p53-mediated apoptosis, competing with p53 for binding to p300 protein. Therefore, macropeptoid **24** can be a chemical probe to define the role of SK2 in tumorigenesis.

Early studies in silico demonstrate how computational tools, using the reported Rosetta suite of protein design algorithms [99], can facilitate the design of macrocyclic peptoid–peptide hybrids. As an example, peptoid **25** (Figure 7) is able to bind to a pocket in β-catenin, involved in the association with T-cell factor (TCF), thus disrupting the β-catenin-TCF interaction [100]. In prostate cell lines, macrocycle **25** displays potent antiproliferative activity, inhibiting both the Wnt- and AR-signaling pathways. This compound also shows in vivo activity in a zebrafish model. 

The group of Hyun-Suk Lim described recently the design and preparation DNA-encoded library (one-bead one-compound format) of cyclic peptoids (>11 million molecules). This library was screened for binding affinity to S-phase kinase-associated protein 2 (Skp2), an adaptor protein involved in cell-cycle progression, senescence, and metabolism. Fluorescence anisotropy assays defined five hit beads, among them, cyclic peptoid **26** (Figure 7) that shows high affinity for Skp2 (K_D_ = 7.51 µM), better than those obtained for the all *N*-methyl analogue and its linear peptoid counterpart [101]. This petoid could be of interest as disruptor of the Skp, Cullin, F-box containing (SCF)-Skp2 complex formation, which has an important role in ubiquitination processes of cell-cycle proteins. Finally, the incorporation of *N*-methylglycine (sarcosine) in transmembrane peptides for the modulation of membrane PPIs has also been studied [102].

## 3. Conclusions and Perspectives

The advantages of cyclic peptides over linear ones, mainly better stability and improved control of the conformational space, has stimulated the interest of numerous research groups in this type of compounds. Due to their high size, compared to small-molecules, cyclic and macrocyclic peptides represent a strategic source in the search for protein–protein interaction (PPI) modulators. In this short review we have tried to illustrate main methods to access this type of molecules, selecting some representative examples of modulators for therapeutically relevant PPIs.

Main advances in computational methods are addressing the complex design of new cyclic peptides, while phage or yeast expression gives rise to large collections of compounds for exploring different PPIs. Organic synthesis is not far behind, guided by the advancement of solid-phase methodologies (SPPS), allocating the preparation of diverse, complex cyclic/macrocyclic peptide libraries. It is also worth highlighting the combination of SPPS with efficient reactions, such as multicomponent processes (mainly those based on cyclic imines), for exploring new chemical space to develop macrocyclic peptides. The cellular permeability of this type of molecules is also being successfully addressed, primarily through the incorporation of cell penetrating peptides CPP), linked either through spacers or condensed into bicyclic structures. The incorporation/combination of β-amino acids and peptoids is also contributing to the expansion of chemical architectures and to obtain metabolically stable systems.

In the time of the dreadful pandemic, cyclic and macrocyclic peptides could also play an important role in the search for antiviral agents. It is now fairly well established that the virus spike protein interacts with ACE2 and neuropilin-1 receptors through protein–protein interactions [103,104,105]. Similarly, the main protease (Mpro) of SARS-CoV-2, the causative etiological agent of COVID-19, is a transient homodimer indispensable for processing virus essential proteins [106]. Investigation is open for peptides and small organic molecules capable of destabilizing the homodimerization and, therefore, for preventing the activity of Mpro.

It is estimated that there could be 650,000 disease-relevant PPIs, but most of them remain unexplored already. Hence, cyclic and macrocyclic peptides can have tremendous potential. Inspiration and creativity of researchers working in this field, theoretical and synthetic chemists, biochemists, molecular biologists, bioinformatics, will contribute to the discovery of new PPIs and to illuminate the generation of different complex peptide structures for disrupting/stabilizing them. They could be novel biological/chemical tools and perhaps new future drugs, hence involved scientists have a long road ahead.
